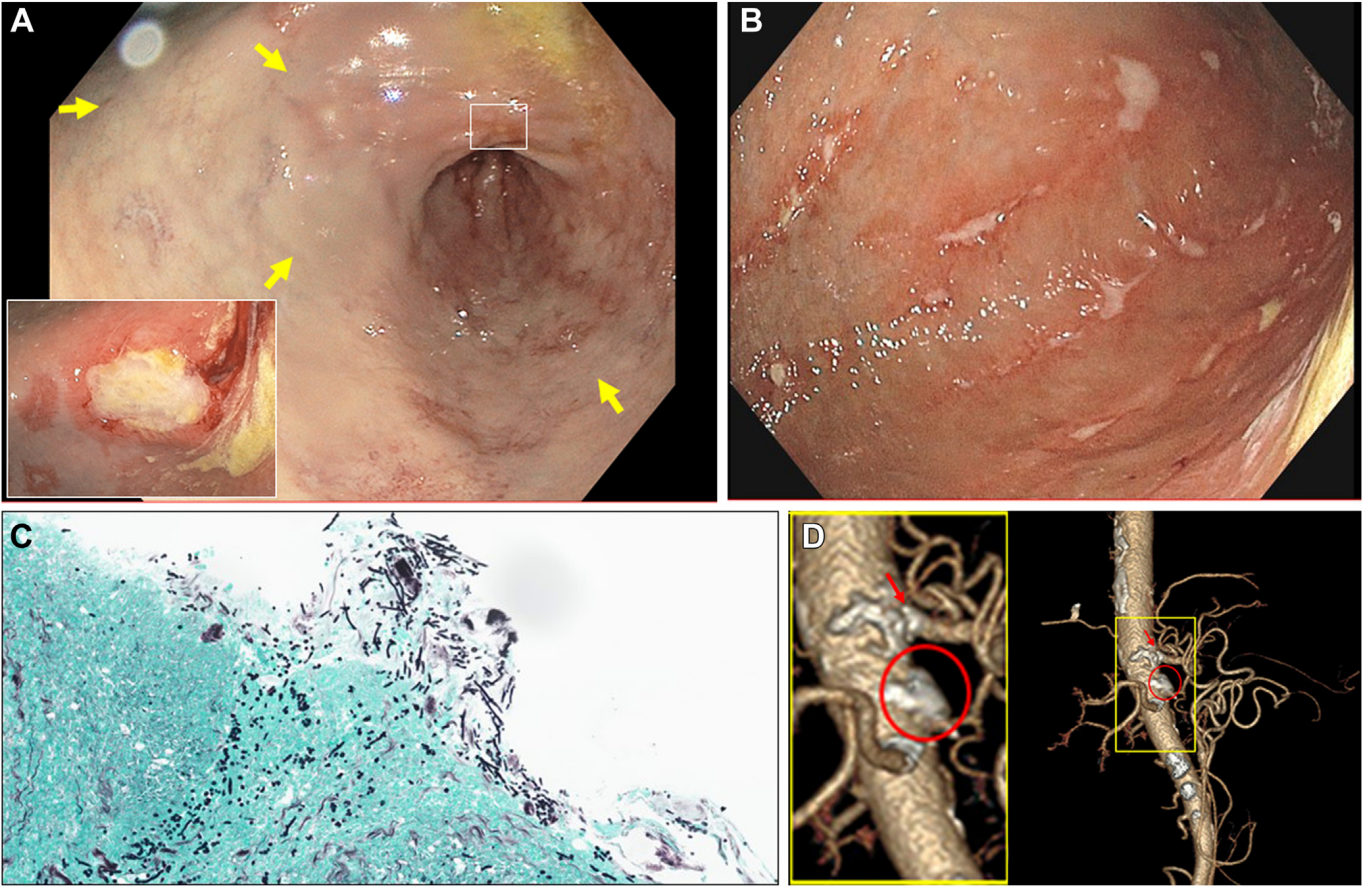# Gastric Ischemia From Partial Dual-Vessel Occlusion

**DOI:** 10.1016/j.gastha.2024.10.013

**Published:** 2024-10-16

**Authors:** Fangfang Wang, Amani Elshaer, Vijay P. Singh

**Affiliations:** 1 Division of Gastroenterology and Hepatology, Mayo Clinic Arizona, Phoenix, Arizona; 2 Department of Medicine, Mayo Clinic Arizona, Phoenix, Arizona

A 76-year-old female with gastroesophageal reflux disease, hypertension, hyperlipidemia, type 2 diabetes, and sick sinus syndrome presented with postprandial epigastric pain and a 68-pound weight loss over one year. Endoscopy revealed a strikingly pale gastric mucosa with cyanotic discoloration (Arrows, Figure A). Body and antrum exhibited erosions and ulcerations in a linear pattern (Figure B). A large incisura ulcer with a firm fibrotic base did not bleed after biopsy (Inset Figure A). Pathology confirmed necrotic ulcer, chronic active gastritis, and dense *Candida albicans* (Figure C). CT angiogram showed occlusion of the proximal superior mesenteric artery (SMA) and high-grade stenosis at the celiac origin (circle, arrow, respectively, in Figure D). The patient underwent SMA stenting with Plavix and fluconazole therapy. These resolved symptoms and a repeat endoscopy in 2 months showed a normal stomach. This case demonstrates gastric ischemia due to partial celiac occlusion and complete SMA occlusion. Postprandial vascular demand caused watershed ischemia and pain despite partial celiac patency. Necrotic, ischemic tissue colonization led to candidiasis. Postprandial pain, weight loss, and endoscopic findings of a pale cyanotic mucosa, with gastric ulcers that bleed minimally on biopsy should prompt vascular imaging. Timely identification and revascularization can improve outcomes.

**Figure:**